# A Fungal Defensin Inhibiting Bacterial Cell-Wall Biosynthesis with Non-Hemolysis and Serum Stability

**DOI:** 10.3390/jof8020174

**Published:** 2022-02-10

**Authors:** Sudong Qi, Bin Gao, Shunyi Zhu

**Affiliations:** 1 Group of Peptide Biology and Evolution, State Key Laboratory of Integrated Management of Pest Insects and Rodents, Institute of Zoology, Chinese Academy of Sciences, 1 Beichen West Road, Chaoyang District, Beijing 100101, China; qisudong@ioz.ac.cn (S.Q.); gaob@ioz.ac.cn (B.G.); 2 University of Chinese Academy of Sciences, Beijing 100049, China

**Keywords:** antimicrobial peptide, Pyronesin4, *Pyronema confluens*, UDP-MurNAc-pentapeptide, Lipid II, molecular dynamics simulation, epistasis

## Abstract

Defensins are a class of cationic disulfide-bridged antimicrobial peptides (AMPs) present in many eukaryotic organisms and even in bacteria. They primarily include two distinct but evolutionarily related superfamilies (*cis* and *trans*). Defensins in fungi belong to the members of the *cis*-superfamily with the cysteine-stabilized α-helical and β-sheet fold. To date, many fungal defensin-like peptides (fDLPs) have been found through gene mining of the genome resource, but only a few have been experimentally characterized. Here, we report the structural and functional characterization of Pyronesin4 (abbreviated as Py4), a fDLP previously identified by genomic sequencing of the basal filamentous ascomycete *Pyronema confluens*. Chemically, synthetic Py4 adopts a native-like structure and exhibits activity on an array of Gram-positive bacteria including some clinical isolates of *Staphylococcus* and *Staphylococcus warneri*, a conditioned pathogen inhabiting in human skin. Py4 markedly altered the bacterial morphology and caused cytoplasmic accumulation of the cell-wall synthesis precursor through binding to the membrane-bound Lipid II, indicating that it works as an inhibitor of cell-wall biosynthesis. Py4 showed no hemolysis and high mammalian serum stability. This work identified a new fungal defensin with properties relevant to drug exploration. Intramolecular epistasis between mutational sites of fDLPs is also discussed.

## 1. Introduction

As the effectors of innate immunity in multicellular organisms, antimicrobial peptides (AMPs) establish the first-line defense in the fight against infections from a variety of microorganisms such as bacteria, fungi, viruses, and protozoa [1,2]. Due to their evolutionary success as host defense molecules across the history of multicellular organisms and less susceptibility to microbial resistance, AMPs have been considered as promising alternatives to conventional antibiotics in the age of resistance [2,3,4]. They are mostly small cationic molecules of <100 amino acids and often bind to an accessible negatively charged microbial membrane via their positively charged residues to cause irreversible membrane damage and cellular death [2,5]. Some AMPs work as a metabolic inhibitor to interact with non-membrane bacterial targets such as Lipid II, outer membrane proteins, and intracellular components (e.g., DNA and proteins) to inhibit their growth [4,6,7]. AMPs markedly differ in sequence and structure and are roughly divided into three major categories on the basis of their amino acid composition and structural types: (1) Linear α-helical AMPs that adopt a helical configuration when contacting microbial membrane, e.g., cecropins from insect hemolymph [8], magainins from frog skins [2], and LL-37 from human neutrophils [9]; (2) Specific amino acid-rich linear AMPs that often lack a defined secondary structure (e.g., PR-39 from pigs [10] and metchnikowin and drosocin from *Drosophila* [11]); and (3) Disulfide-containing AMPs with a distinct structure stabilized by disulfide bridges (e.g., Protegrin-1 (PG-1) from pigs [12] and defensins from plants, animals, and humans [13]. In addition, a minor category of AMPs is the cyclic peptides with a head-to-tail cyclized architecture (e.g., bacteriocins from bacteria) [2].

Defensins are a group of antimicrobial and cytotoxic peptides first identified in 1985 from human neutrophils [14] and are now recognized as part of a large superfamily of *trans*-defensins from vertebrates including α-, β-, and θ-defensins, and the bi-domain big defensins from invertebrates [13,15]. Another superfamily of defensins called *cis*-defensins are found in plants, invertebrates (e.g., insects, scorpions, and mussels), fungi, and bacteria [13,16,17,18,19]. Unlike *trans*-defensins that have a common β-sheet core stabilized by three disulfide bridges [20], *cis*-defensins adopt a unique cysteine-stabilized α-helical and β-sheet (CSαβ) fold with a conserved disulfide linkage pattern [17]. Evolutionarily, the *cis*- and *trans*-defensins might originate from a common ancestor [21]. These *cis*-defensins can be further divided into three subgroups based on their sequence similarity: antibacterial ancient invertebrate-type defensins (AITDs), antibacterial classical insect-type defensins (CITDs), and antifungal plant/insect-type defensins (PITDs) [22].

Plectasin is the first fungal defensin found in *Pseudoplectania nigrella*, which can be considered as the prototype of fungal defensin-like peptides (fDLPs) [16]. It kills sensitive bacteria via a mode analogous to the lantibitoic nisin [23], although nisin inserts deeply into the membrane bilayer to form pores [24]. In this mode, the two terminal loops of plectasin (designated as N-loop and C-loop) contribute most of the hydrophobic residues to dock itself to the bacterial membrane (herein called membrane contact region) and structurally adjacent residues derived from the N-terminus and the secondary structure elements bind Lipid II (herein called Lipid II binding region) to inhibit bacterial cell-wall biosynthesis [24]. Since its discovery in 2005 [16], many plectasin homologs have been found in multiple fungi Phyla such as Ascomycota, Zygomycota, Basidiomycota, and Glomeromycota through comparative genomics analysis [25]. These fDLPs can be assigned into the three subgroups of *cis*-defensins in animals and plants, of which only a few members are structurally and functionally identified. Of them, only those with sequence and structure closely resembling the animal derived AITDs showed antibacterial activity (e.g., eurocin and micasin) [22,26,27].

In this work, we computationally and experimentally evaluated Pyronesin4 (abbreviated as Py4), an fDLP previously identified by genomic sequencing of the basal filamentous ascomycete *Pyronema confluens* (also known as *Pyronema omphalodes*) [25] in terms of its sequence, structural and dynamics features, antibacterial activity, action mode, and therapeutic potential relevant properties. Py4 is a natural variant of plectasin with two unique point mutations optimized in evolution via epistasis. Experimental investigations indicate that Py4 is a promising anti-infective molecule with high serum stability and no hemolysis. The potent antibacterial activity on clinical isolates of *Staphylococcus* and *S. warneri*, a conditioned pathogen inhabiting in human skin, makes it a candidate for further exploration into an anti-*Staphylococcus* drug.

## 2. Materials and Methods

### 2.1. Construction of Py4-Lipid II Complex

The model structure of Py4 was obtained by introducing two point mutations (Q14K and G33A) into the plectasin structure (pdb entry 1ZFU) with Swiss-PdbViewer 4.1.0 followed by energy minimization with the GROMOS96 force-field in vacuo without the reaction field (https://spdbv.vital-it.ch/, accessed on 20 April 2021). This structure was used to dock with Lipid II, which is complexed with plectasin [24] by a template-based docking method [28]. The initial Py4-Lipid II complex was optimized by energy minimization using the Amber12: EHT force-field implemented in Molecular Operating Environment (MOE) 2019.0102.

### 2.2. Molecular Dynamics (MD) Simulations

For comparison purposes, 50 ns of MD simulations was performed for Py4 and plectasin with the GROMACS 2020.1 software package (http://www.gromacs.org/, accessed on 20 April 2021). The force-field used was the OPLS (Optimized Potential for Liquid Simulations)-AA/L all-atom force-field (2001 amino acid dihedrals) and TIP3P model for explicit water. Solvent shell thickness was 1.0 nm in a cubic box and the total charge of the simulated systems were neutralized by adding sodium or chloride ions. The system was subsequently equilibrated with NVT (number of particles, volume, and temperature) and NPT (number of particles, pressure, and temperature), in which all protein atoms were restrained to their initial position. Following these two equilibration stages, production MD was performed according to the method previously described [29]. Root-mean-square deviation (RMSD) and root-mean-square fluctuation (RMSF) were calculated with the trajectory analysis tools implemented in GROMACS 2020.1.

### 2.3. Identification of Chemically Synthesized Py4

Py4 was chemically synthesized by Zhejiang Ontores Biotechnologies Co., Ltd. (Hangzhou, China) and its oxidized form was produced by air oxidization in an alkaline solution of *N*,*N*-diisopropylethylamine (pH8.0), which was further purified by reverse phase-high performance liquid chromatography (RP−HPLC). Its molecular weight (MW) was determined by MALDI−TOF (matrix-assisted laser desorption/ionization time of flight) mass spectrometry performed on MALDI−TOF/TOF Ultraflextreme^TM^ (Bruker Daltonics, Bremen, Germany) using α-acyano-4-hydroxycinnamic acid (α-CHCA) as the matrix in the positive-reflection ion mode.

### 2.4. Circular Dichroism (CD)

The secondary structure of Py4 was studied by CD spectroscopy analysis on a Chirascan Plus spectropolarimeter v.4.4.0 (Applied Photophysics Ltd., Leatherhead, Surrey, UK). The spectra were measured at room temperature from 185–260 nm by using a quartz cell of 1.0 mm thickness and the spectra from 190–260 nm were analyzed. Data were collected at 1 nm intervals with a scan rate of 2 nm/s and expressed as delta epsilon (cm^−1^M^−1^) calculated as [θ × (MRW × 0.1)/(C × L)/3298], where θ is the ellipticity (in millidegrees), C is the concentration (in mg/mL), L is the pathlength (in cm), and MRW is the mean residue weight (in Da).

### 2.5. Antibacterial Assay

The inhibition-zone assay was used to quantify the antibacterial activity of Py4, as described previously [8,19]. In brief, an overnight bacterial culture from a single colony was inoculated into fresh medium and grew to the late log-phase. A 10 μL aliquot of each culture was diluted in 6 mL pre-heated medium containing 0.8% agar. The mixture was spread on a 9-cm Petri dish, giving a depth of 1 mm. After settling, 3-mm wells were punched in the plate and then 2 μL peptide samples of different concentrations were added to each well. The agar plates were incubated overnight at indicated temperatures. A lethal concentration (C*_L_*) was calculated from a plot of d^2^ against log n, where d is the diameter (in cm) and n is the amount of sample applied in the well (in nmol). The plot is linear and thus C*_L_* can be calculated from the slope (k) and the intercept (m) of this plot. The formula used here was C*_L_* = 2.93/ak10^m/k^, where a is the thickness of the bacterial plate and C*_L_* is in μM. In most cases, the calculated C*_L_* were comparable with the directly measured minimal inhibitory concentrations (MIC) via the broth micro-dilution assay [30].

### 2.6. Membrane Permeability Assay

The membrane permeability assay was performed according to the method previously described [19]. Meucin-18 and Meucin-49, two known scorpion venom lytic peptides [31,32], were used as positive controls and vancomycin, a known bacterial cell-wall synthesis inhibitor without membrane-disruptive ability [19] as a negative control. *Bacillus megaterium* cells in 500 μL of phosphate buffered saline (PBS) (pH 7.3) were mixed with 1 μM propidium iodide (PI) for 5 min in the dark. Fluorescence was measured with the F-4500 FL spectrophotometer (Hitachi High-Tech Science Corporation, Tokyo, Japan). Once the basal fluorescence reached a constant value, peptides or vancomycin at 10 × C*_L_* were added, and changes in fluorescence arbitrary were monitored (λ_exc_ = 525 nm; λ_ems_ = 595 nm).

### 2.7. Scanning Electron Microscope (SEM)

*Curtobacterium luteum* cells at the exponential growth phase were treated with 20 × C*_L_* (0.6 μM) of Py4 or 20 × vancomycin (2.0 μM) at 37 °C for 90 min. After centrifugation, bacterial pellets were fixed with 2.5% glutaraldehyde at 4 °C overnight, followed by washing three times with water. Dehydration was carried out with a series of graded ethanol solutions. Cells were then dried by a Leica EM CPD300 Automated Critical Point Dryer (Leica Microsystems, Wetzla, Germany) before being mounted on carbon tape and sputtered with a gold coating by a Leica EM SCD050. Images were visualized by using a FEI Quanta 450 scanning electron microscope (FEI, Hillsboro, OR, USA).

### 2.8. Cytoplasmic Accumulation of UMP

The cytoplasmic UDP-MurNAc-pentapeptide (abbreviated as UMP) accumulation experiment was carried out according to the method previously described [19,33]. In brief, *S. warneri* cells were grown at 37 °C to an OD_600_ of 0.5 in 40 mL broth medium and then the culture was supplemented with 130 μg/mL chloramphenicol to impede bacterial cell proliferation by blocking their protein synthesis. After 15 min of incubation, the culture was divided into three equal portions, and one portion was treated with Py4 (0.6 μM), the second with vancomycin (2.0 μM), and the third was used as an untreated control. UMP was extracted from the harvested cells following 30 min of incubation. UMP was identified by MALDI−TOF on MALDI−TOF/TOF Ultraflextreme^TM^ (Bruker Daltonics, Bremen, Germany) using 2,5-dihydroxybenzoic acid (DHB) as the matrix in the negative-reflection ion mode.

### 2.9. Electrospray Ionization Mass Spectrometry (ESI-MS)

Bio-affinity electrospray ionization mass spectrometry (ESI-MS) was used to study non-covalent interactions between Py4 and the *S. warneri* cell wall precursor—UMP according to the method previously described [19]. All components for interactions were dissolved in 25 mM ammonium acetate (pH5.2). Electrospray spectra were recorded with a Waters SynaptG2-Si mass spectrometer (Waters, Manchester, UK) and measurements were performed with positive ESI in resolution mode. Ions were scanned in a mass range of 500 to 2000 *m*/*z* with the following conditions: the capillary voltage at 1500 V; the cone voltage at 40 V; and the source block temperature at 30 °C. Spectra were recorded and processed by the software Masslynx4.1 (Waters, Manchester, UK).

### 2.10. Hemolysis Assays and Stability

Hemolytic activity of peptides against fresh erythrocytes from ICR mice (*Mus musculus*) was assayed according to the standard method [19]. Peptides were diluted with 0.9% NaCl to the indicated concentration. The percentage of hemolysis is determined as (A_pep_ − A_blank_)/(A_tot_ − A_blank_) × 100, in which “A” represents absorbance measured at 570 nm. A_blank_ and A_pep_ were evaluated in the absence or presence of peptides. One hundred percent hemolysis (A_tot_) was obtained in the presence of 1% Triton X-100. For the stability assay, Py4 was incubated in H_2_O or fresh mouse serum for the indicated times at 37 °C and their residual activity was measured by the inhibition-zone assay [19].

## 3. Results

### 3.1. Py4 Has Two Unique Point Mutation Sites and Exhibits a More Rigid Structure than Plectasin

**Figure 1:**
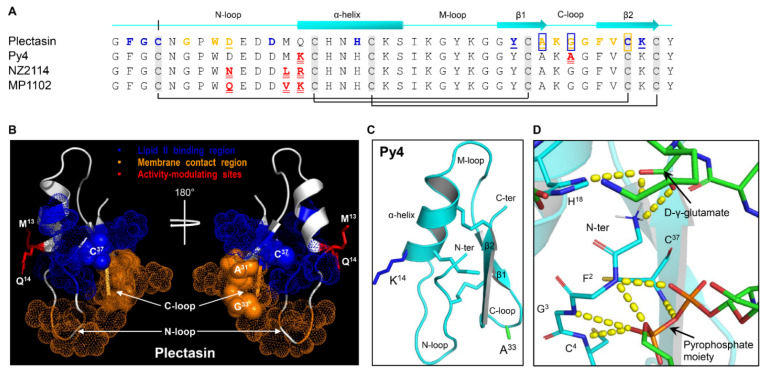
Plectasin and its natural variant Py4. (**A**) Sequence comparisons with two engineered mutants (NZ2114 and MP1102) included. Secondary structure elements (the cylinder for the α-helix and arrows for β-strands), loops and disulfide bridges are drawn according to the coordinates of plectasin (pdb entry 1ZFU). Residues of plectasin involved in interacting with Lipid II and bacterial membrane are shown, respectively, in blue and orange, with those implicated in both boxed [24]. Residues whose mutations diminished the activity are underlined once [24]. Mutational sites in Py4 and the engineered peptides are colored in red and underlined twice. (**B**) The functional surface of plectasin. Residues belonging to the Lipid II binding and membrane contact regions are shown by dots in blue and orange, respectively, with those belonging to both shown by spheres. Activity-modulating sites whose mutations improved the activity but not involved in Lipid II and membrane binding [24,34,35] are shown as red sticks. (**C**) The Py4 structure. The two mutation residues are shown as sticks in different colors. Secondary structure elements and three intercysteine loops are labeled. (**D**) Structural basis of Py4 interacting with Lipid II. The complex was built by template-based molecular docking from the plectasin-Lipid II complex. Indicated in yellow dashed lines are hydrogens in the complex, in which involved amino acid residues and pyrophosphate moiety are labeled.

Compared with plectasin, the natural variant Py4 has two point mutations at positions 14 and 33 (Figure 1A) that are respectively located on the α-helix (Q14K) and the C-loop (G33A) (Figure 1A). These two mutations are interesting considering their mutational effects previously reported in plectasin. (1) Position 14. This site is not involved in direct interactions with both Lipid II and bacterial membrane [24], but its mutation from the polar Qln to a cationic amino acid (Arg or Lys) conferred enhanced antibacterial activity in two engineered plectasin mutants (NZ2114 and MP1102) [34,35], suggesting that it belongs to a class of activity-modulating sites (Figure 1A). (2) Position 33. This site is rather unique since it exerts NMR (nuclear magnetic resonance) chemical shift-changes both upon the addition of the membrane mimicking dodecylphosphocholine (DPC) micelles and Lipid II [24], highlighting its key role in conferring to both Lipid II and membrane binding (Figure 1A,B). Its functional importance is further strengthened by site-saturated mutagenesis, which showed that no amino acid substitutions at this position (Gly-33) resulted in activity against *S. aureus* [24], indicating that this glycine is indispensable for the antibacterial function of plectasin. Intriguingly, in Py4, this position is occupied by an alanine instead of a glycine. In such a case, there would be two possibilities in terms of its activity: (1) Py4 would lack antibacterial activity due to the mutation, as observed in the plectasin Gly-33 mutants [24]; and (2) if Py4 shows antibacterial activity, this suggests that the accompanying mutation at position 14 (Q14K) creates a new genetic background different to that of plectasin by epistasis [36] to maintain the activity.

Using homology modeling combined with template docking, we built a theoretic complex between Py4 and Lipid II based on that of plectasin and Lipid II (Figure 1C,D). Analysis of the complex structure revealed that the conserved hydrogen bond network mediating the interaction of plectasin with Lipid II [24] also exists in the Py4-Lipid II complex. The residues involved in the network include the N-terminus, His-18, Phe-2, Gly-3, Cys-4, and Cys-37 of Py4 and the D-γ-glutamate and the pyrophosphate moiety of Lipid II (Figure 1D), suggesting that Py4 would also target Lipid II if it has an antibacterial activity.

**Figure 2:**
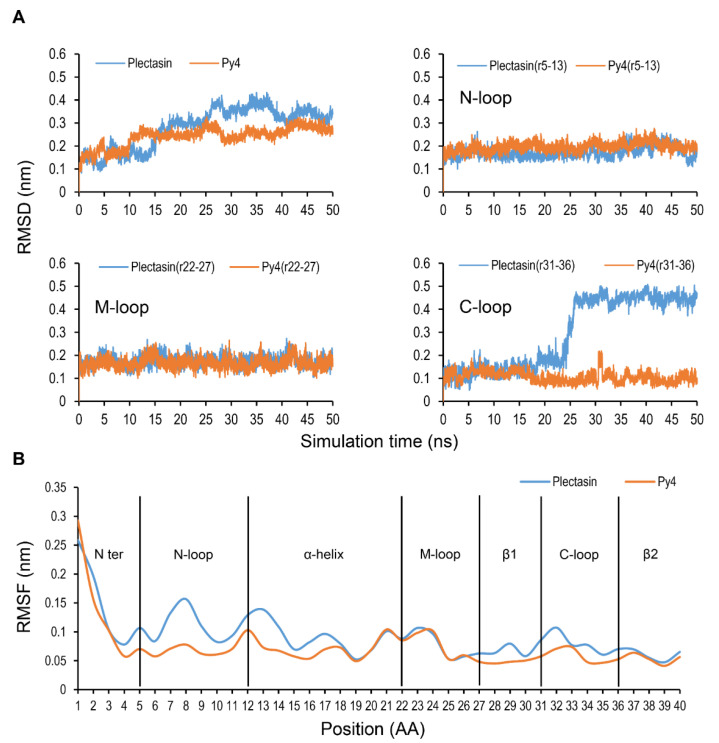
Comparison of the molecular dynamics behavior between Py4 and plectasin. (**A**) RMSD values of Py4 and plectasin as well as their three loops during 50-ns MD simulations. (**B**) RMSF values calculated for the Cα atoms per residue in Py4 and plectasin. Indicated in vertical lines are the secondary structure traits extracted from the NMR structure of plectasin.

To investigate the potential structural effects of the mutations on Py4, we carried out molecular dynamics (MD) simulations to compare changes in structural stability and flexibility between Py4 and plectasin. The results showed that after 25 ns of simulations, their root-mean-square deviation (RMSD) curves began to deviate remarkably from each other over time (Figure 2A). Further analyses of individual loops revealed that Py4 and plectasin both have similar RMSDs in the N-loop (residues 5–12) and the M-loop (residues 22–27) whereas larger deviation occurred after 25-ns simulations in the C-loop (residues 31–36) (0.1 nm in Py4 and 0.4 nm in plectasin) (Figure 2A). This indicates that this loop essentially contributes to the mutation-elicited structural deviation. In plectasin, this loop connects the C-terminal β2 and β3 and is a functional region involved in antibacterial activity via interacting with both bacterial membrane and Lipid II [24]. In Py4, this loop exhibited an enhanced structural stability than plectasin, which could be could be a consequence of the G33A mutation since glycine is a unique amino acid without the beta carbon, is usually flexible, and can take on polypeptide backbone conformations. This mutation definitely leads to an increased structural stability in the loop of Py4. RMSF analysis revealed that several positions, particularly the N-loop and the α-helix of Py4, exhibited smaller fluctuation compared with plectasin (Figure 2B), indicative of the mutation-evoked conformational changes occurring at the adjacent and remote regions of the two mutational sites.

### 3.2. Chemically Synthetic Py4 Adopt a CSαβ-Type Structure with Potent Antibacterial Activity

**Figure 3:**
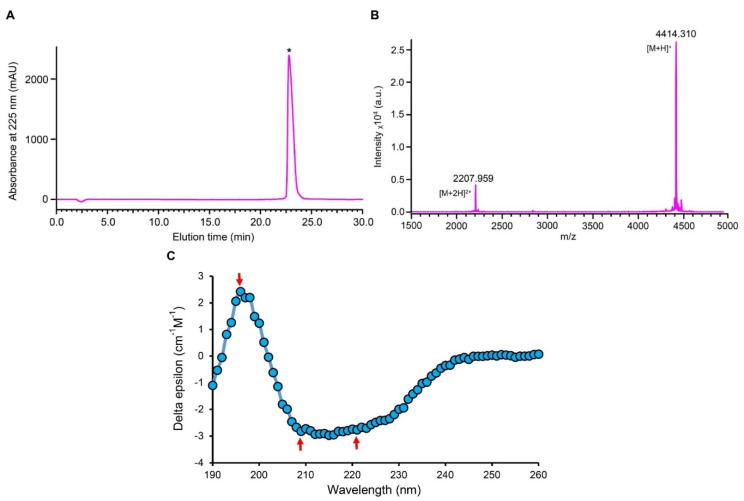
Characterization of chemically synthesized Py4. (**A**) RP−HPLC profile. The Agilent Zorbax 300SB-C18 (4.6 × 150 mm, 5 μm) was equilibrated with 0.05% TFA in water (*v*/*v*) and peptides were eluted from the column with a linear gradient from 0 to 60% acetonitrile in 0.05% TFA within 40 min with a flow rate of 1 mL/min. The UV absorbance was monitored spectrophotometrically at 225 nm. The star marks the elution peak of Py4. (**B**) MALDI−TOF MS. The two main peaks corresponded to the singly and doubly protonated forms, respectively. (**C**) CD spectroscopy analysis of Py4. The peptide concentration used was 0.13 mg/mL. The distinct CD spectroscopic signatures for secondary structural elements are indicated by red arrows.

To experimentally investigate the structural and functional features of Py4, we used the oxidized peptide refolded from its chemically synthesized linear peptide with a purify >95%, as identified by RP−HPLC with a C_18_ analytical column and MALDI−TOF (Figure 3A,B). The experimental m/z of 4414.310 well matched the calculated molecular weight of 4416.0 Da, indicating that the refolded Py4 formed three disulfide bridges. CD spectroscopy analysis showed that Py4 had two negative bands at 209 nm and 221 nm, respectively, and a positive band at 196 nm (Figure 3C). These absorbance minima and maxima respectively fell within the scope of spectroscopic signatures of the α-helix [i.e., 207–210 nm (−) and 221–222 nm (−)] and β-sheet [i.e., 195–197 nm (+)], indicating that the refolded Py4 adopts a native-like CSαβ structure.

Using the classical inhibition-zone assay, we evaluated the antibacterial activity of Py4 referring to the antibacterial spectrum of plectasin [16]. The results can be summarized as follows (Table 1): (1) Py4 exhibited an excellent antibacterial activity on *Curtobacterium luteum*, a psychrotrophic, plant growth-promoting endophytic bacterium [37,38], with a C*_L_* of 0.03 μM, and the endospore forming bacterium *B. megaterium* with a C*_L_* of 0.67 μM. (2) Py4 is also highly effective on *Staphylococcus warneri*, a coagulase negative *Staphylococcus* found in human skin flora [39] with a C*_L_* of 1.06 μM. (3) Py4 showed a moderate to strong activity on other *Staphylococcus* species, with a C*_L_* range between 1.22 and 4.78 μM. These species include several antibiotic-resistant clinical isolates (e.g., penicillin-resistant *Staphylococcus epidermidis* P1389; methicillin-resistant *Staphylococcus aureus* P1374; methicillin-resistant coagulase negative *Staphylococci* P1369 and methicillin-resistant *Staphylococcus aureus* P1386) [19,27] (Table 1). (4) Py4 exhibited no activity on *Escherichia coli* at the concentration range used here.

### 3.3. Py4 Inhibits Bacterial Cell-Wall Biosynthesis

**Figure 4:**
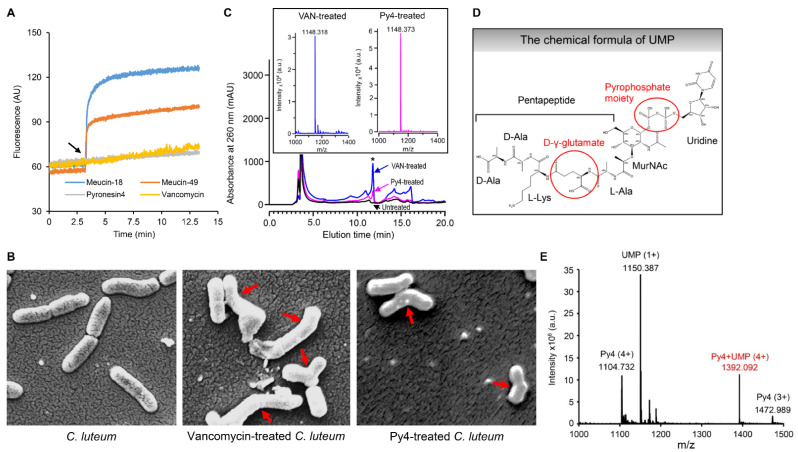
Evidence for cell wall synthesis inhibition by Py4. (**A**) The effect of Py4 on the *B. megaterium* membrane at a 10 × C*_L_* dose. The dead cell stain propidium iodide was used to monitor the ability of membrane damage by Py4 as a function of time based on its nature, which emits red fluorescence when bound to DNA, in which vancomycin and Meucin-18 and Meucin-49 [18,32] were used as negative and positive controls, respectively. The arrow denotes the time point for drug addition. (**B**) Scanning electron microscopic observation of Py4-induced *C. luteum* deformation. Left, *C. luteum* without peptides. Middle, Vancomycin-treated *C. luteum*. Right, Py4-treated *C. luteum*. Red arrows indicate cells with an obvious morphological change. For their full images, see Figure S1. (**C**) Cytoplasmic accumulation of the soluble cell-wall precursor UMP shown by RP−HPLC analy-sis of the water-soluble components from the culture of S. warneri cells treated by Py4 (pink line) or vancomycin (blue line) or not treated (black line). The star marks the elution peak of UMP. (In-set) MALDI−TOF identifying UMP from vancomycin-treated cells (blue) and Py4-treated cells (pink). (**D**) The chemical formula of UMP. The components involved in interactions with defensins are circled in red. (**E**) ESI-MS detecting the non-covalent complex between Py4 and UMP. The quadruply protonated complex (*m/z* 1392.092) shown in red was observed. The components detected included 2.5 μL of 500 μM Py4; 2.5 μL of 500 μM *S. warneri* UMP and 5 μL of 50 mM NH_4_OAc (pH 5.2).

Using the fluorescence dye—propidium iodide (PI), we assessed the membrane-disruptive activity of Py4 on *B. megaterium* by monitoring DNA release. The results showed that the addition of this peptide caused no obvious fluorescent increase, as observed in the cells treated by vancomycin, an antibiotic inhibitor of bacterial cell wall synthesis (Figure 4A). Conversely, two classical membrane-active AMPs (Meucin-18 and Meucin-49) evoked such an effect. This experimental result indicates that Py4 does not kill bacteria through disrupting their membrane integrity. Subsequently, we used SEM to observe the impact of Py4 on the cellular morphology of *C. luteum*. We found that the untreated *C. luteum* cells grew with a straight rod shape whereas some treated cells by Py4 became curved rods (Figure 4B, see Figure S1 for the full image). A similar phenomenon was also observed in the vancomycin-treated cells (Figure 4B and Figure S1), suggesting that this peptide may work in a similar manner to the inhibitors of cell-wall synthesis.

We obtained further evidence for this inference via analysis of the intracellular pool of *S. warneri* cell-wall synthesis precursors. By means of RP–HPLC, we identified a common emerging peak derived from the intracellular soluble molecules from both Py4- and vancomycin-treated cells, which was totally absent in the untreated cells (Figure 4C). MALDI–TOF mass spectrometry analysis confirmed that this accumulated soluble molecule was UDP-MurNAc-pentapeptide (abbreviated as UMP) (Figure 4C,D). Since UMP shares a pyrophosphate group and the γ-glutamic acid with Lipid II for interacting with the fungal and bacterial defensins (i.e., plectasin and AMSIN) [19,24], we used UMP as an alternative of Lipid II to study the interaction of Py4 with the cell-wall precursor. Using bio-affinity ESI-MS, a technique for the evaluation of solution-phase non-covalent interactions that has succeeded in the detection of the complex of bacterial defensing, AMSIN and UMP [19,40], we detected a quadruply protonated complex between Py4 and UMP at *m*/*z* 1392.092 (Figure 4E). This implies that this peptide kills sensitive bacteria via inhibiting their cell-wall biosynthesis.

### 3.4. Py4 Is a Non-Hemolytic Defensin with Serum Stability

**Figure 5:**
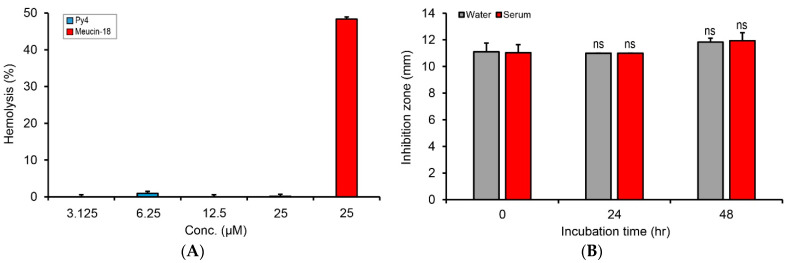
Evaluation of hemolysis and stability of Py4. (**A**) No hemolysis on mouse blood cells by Py4. Meucin-18, a scorpion venom-derived lytic peptide [18], was used as the positive control. (**B**) The inhibition zone-based assay for evaluating the stability of Py4 in water and mouse serum. The bacterium used was *B. megaterium* and the peptide dose was 0.2 nmol/well. Independent-samples T-test was used to compare the means between the control (0 h) and treatment groups (24 and 48 h) with SPSS (SPSS Inc.). “ns”, no significance.

To explore the potential therapeutic value of Py4, we evaluated its two important properties, namely hemolysis and serum stability, which are highly relevant to the therapeutic efficacy of a peptide drug. In the hemolysis assay, we found that Py4 showed no significant hemolysis on mouse blood cells at a concentration range from 3.125 to 25 μM using 2-fold serial dilutions. In contrast, the positive control peptide—Meucin-18—showed strong hemolysis at 25 μM (Figure 5A). In the serum stability assay performed using the inhibition-zone method to determine the effect of incubation in water or serum on the antibacterial activity, we found that Py4 retained its full antibacterial activity after 24- and 48-h incubation in both water and the undiluted fresh mouse serum at 37 °C (Figure 5B). These data indicate that Py4 is a non-hemolytic defensin with high mammalian serum stability.

## 4. Discussion

**Figure 6:**
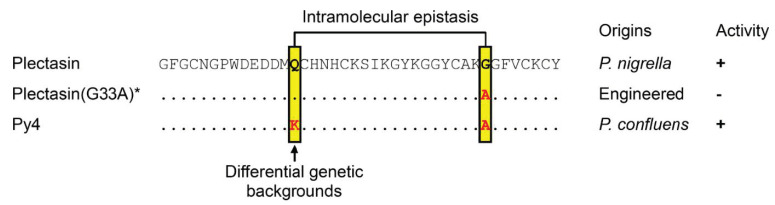
Evidence for intramolecular epistasis in fDLPs. Small dots represent identical residues to plectasin. Positions showing differences in the peptides are boxed and the mutated residues relative to plectasin are colored in red. Positions involved in intramolecular epistasis are linked by one line. The arrow points to the position accounting for differential genetic backgrounds between plectasin and Py4. The “*” denotes that besides the mutant G33A, other non-glycine mutants at this position also showed no activity [24]. “+”, activity; “-“, no activity”.

In this work, we report on the structural and functional characterization of a new fDLP (Py4). By using the chemically synthetic peptide, we confirmed that Py4 adopts a CSαβ structure with activity against multiple Gram-positive bacteria. These results are perhaps not unexpected given that Py4 has only two point mutations compared with plectasin (Figure 1A) and notably, the residues previously identified to be involved in a direct contact of plectasin with the bacterial Lipid II [24] are completely conserved in Py4. However, as mentioned previously, mutations at position 33 from the glycine to any non-glycine amino acids including alanine led to the activity loss of plectasin, implying that the alanine or any other non-glycine residues is deleterious in this genetic background, restraining its mutation. Conversely, in Py4, the G33A mutation does not diminish its antibacterial activity, implying that an alanine at this position is allowable. This raises an interesting evolutionary question regarding how the plectasin otholog Py4 can remove such mutational restraint to maintain its antibacterial activity after species divergence. Our prior study has found that an internal epistatic interaction in Crem-5, a nematode-sourced antifungal defensin with a similar structure to that of Py4, may exert a role in restraining the evolution of its certain site [41]. Such restraint is removed in its otholog Clat-5 through introducing one mutation at another site to relieve the epistatic control [41]. We believe that this also likely occurs in the evolution of Py4. In this evolutionary scenario, the Q14K mutation introduces a new genetic background into the peptide, permitting the occurrence of G33A so that the antibacterial activity remains (Figure 6). This kind of intramolecular epistasis has been found to be able to exert a strong influence on trajectories of protein evolution [42].

Such mutations described above clearly change the dynamics behavior of plectasin and Py4 (Figure 2). In particular, the G33A mutation significantly increased the rigidity of the molecule. This could explain the activity loss in the Gly-33 mutants of plectasin since rational design of peptides has indicated that a more rigid defensin structure is often associated with a lower activity [43]. However, we found that although Py4 has a more rigid structure, it exerts an overall comparable anti-*S. aureus* activity with plectasin [16] (Table 1). This may be explained by the compensatory mutation at position 14 (Q14K) (Figure 6). In line with this explanation, engineering of a Lys into this position has been found to exert a positive role on the antibacterial activity of plectasin. This could compensate for the loss caused by the G33A mutation-evoked rigidity increase in Py4 and thus allows its occurrence in Py4, as previously mentioned. Although the ecological significance of the mutations to its fungal host and related physical interaction between the two positions remain to be established, it appears to be clear that even minor mutations in fDLPs could be of certain evolutionary and functional significance given their occurrence in a new environment following species divergence [16,25,44]. Therefore, for a newfound fDLP even with few mutations with the known ones, it may still be worth further investigation if we consider their new genetic background, likely conferring new values as templates for protein engineering.

A key challenge in the development of AMPs as antimicrobials is their hemolytic effect on human and mammalian erythrocytes, which clearly stems from their fundamental detergent-like properties causing membrane disruption and cellular toxicity [45,46,47,48]. This has been seen in many AMPs such as Melittin and the Meucin series [31,32,48,49]. In addition, many AMPs are unstable when incubated in human and mammalian serums. For example, the antimicrobial activity of human AMPs is inactivated by both human and fetal bovine serums [50,51,52]. This might be related to their physiological roles being limited to the defense of local microenvironment other than delivery to the systemic circulation [19,47]. Conversely, Py4 has two striking properties that are superior to these AMPs: (1) It is of high stably in undiluted mouse serum where it retains full antibacterial activity for a long time; and (2) Py4 is a non-hemolytic peptide. This can be explained by the lack of an overall amphiphilic architecture in Py4, making it impossible to disrupt the bacterial membrane.

Of the Py4-sensitive *Staphylococcus*, *S. warneri* is a commensal skin bacterium that is an opportunistic pathogen that can cause serious diseases in humans such as native valve endocarditis, ventriculoatrial shunt and ventriculoperitoneal shunt infections, meningitis, botryomycosis, urinary tract infection, multifocal discitis, septic arthritis, and vertebral osteomyelitis [53,54,55,56,57,58,59,60,61,62,63,64,65]. Therefore, in addition to the potential value of Py4 against the clinical isolates of *Staphylococcus*, its potent activity on *S. warneri* by targeting the cell-wall biosynthesis should be further explored.

## 5. Conclusions

As a natural variant of plectasin, Py4 exhibits a unique dynamic behavior unlike the prototypical fungal defensin and shows excellent antibacterial activity, particular-ly on some *Staphylococcus* species. Compared with many animal- and human-derived AMPs, Py4 shows two important properties relevant to its therapeutic efficacy, that is, no toxicity on mammalian erythrocytes and high stability in undiluted mammalian serum. In addition, similar to other fungal defensins, Py4 binds to the bacterial cell-wall intermediate Lipid II to achieve its antibiotic effect, a mechanism that may reduce and delay the occurrence of bacterial resistance [19]. All these findings imply that Py4 is a promising candidate for developing into an anti-*Staphylococcus* drug. The discovery that few point mutations can cause a significant structural dynamics change in fungal defensins highlights the importance of the work on continuous mining of fDLPs from the fungal genome resource and experimental evaluation of their activity and action modes through the methods described here, which should be encouraged. This will offer a platform for the future development of lead antimicrobials in the age of resistance. Finally, structural and functional studies of newfound fDLPs will help to shed light on how evolution has shaped their mutations in the context of epistasis.

## Figures and Tables

**Table 1:** Lethal concentrations (C*_L_*) of Py4 against various bacteria.

Strains	C*_L_* (µM)
*Bacillus megaterium* CGMCC 1.0459	0.67
*Curtobacterium luteum*	0.03
*Staphylococcus aureus* CGMCC 1.89	1.22
Penicillin-sensitive *Staphylococcus epidermidis* P1111	4.03
Penicillin-resistant *Staphylococcus epidermidis* P1389	4.38
Methicillin-resistant *Staphylococcus aureus* P1374	2.69
Methicillin-resistant coagulase negative *Staphylococci* P1369	4.78
Methicillin-resistant *Staphylococcus aureus* P1386	2.51
*Staphylococcus aureus* J685	2.69
*Staphylococcus aureus* J698	2.69
*Staphylococcus aureus* J706	2.51
*Staphylococcus warneri* CGMCC 1.2824	1.06

Note: C*_L_* was determined by the inhibition-zone assay.

## Data Availability

All relevant data are within the manuscript.

## Supplementary Materials

The following supporting information can be downloaded at: www.mdpi.com/article/10.3390/jof8020174/s1, Figure S1: Scanning electron microscopic observation of Py4-induced *C. luteum* deformation.

## Abbreviations

3D: three–dimensional; α-CHCA, α-acyano-4-hydroxycinnamic acid; AITD, ancient invertebrate-type defensin; AMP, antimicrobial peptide; CD, circular dichroism; CITD, antibacterial classical insect-type defensin; C*_L_*, lethal concentration; CSαβ, cysteine-stabilized α-helical and β-sheet; fDLP, fungal defensin-like peptide; MALDI–TOF MS, matrix-assisted laser desorption/ionization time of flight mass spectrometry; MW, molecular weight; OPLS, optimized potential for liquid simulations; PG-1, protegrin-1; PBS, phosphate buffered saline; PI, propidium iodide; PITD, antifungal plant/insect-type defensin; Py4, Pyronesin4; RMSD, root-mean-square deviation; RMSF, root-mean-square fluctuation; RP–HPLC, reverse phase-high performance liquid chromatography; SEM, scanning electron microscope; UMP, UDP-MurNAc-pentapeptide.
