# Supplementary material for: A Fungal Defensin Inhibiting Bacterial Cell-Wall Biosynthesis with Non-Hemolysis and Serum Stability
Source: J Fungi (Basel). 2022 Feb 10;8(2):174. doi: 10.3390/jof8020174 (PMC8877149; doi:10.3390/jof8020174)
Supplement: Supplementary file 1 [file jof-08-00174-s001.zip › jof-1562398-supplementary.pdf]

Supplementary Material

Supplement to: Sudong Qi, Bin Gao and Shunyi Zhu. A Fungal Defensin Inhibiting Bacterial Cell-Wall Biosynthesis with Non-Hemolysis and Serum Stability.

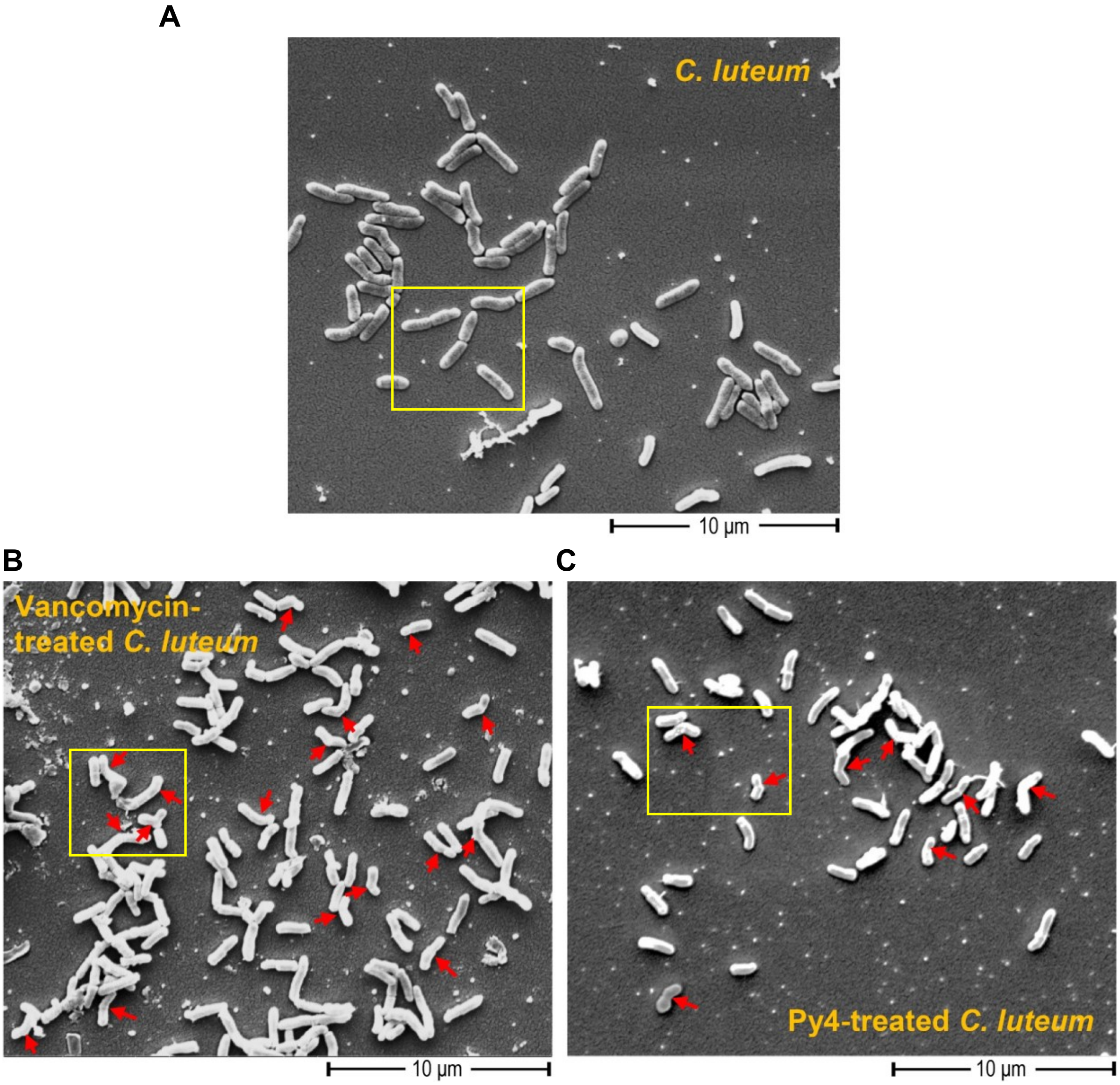

Figure S1. Scanning electron microscopic observation of Py4-induced *C. luteum* deformation. (**A**) *C. luteum* without peptides. (**B**) Vancomycin-treated *C. luteum*. (**C**) Py4-treated *C. luteum*. Red arrows indicate cells with an obvious morphological change. Zoomed-in views appeared in Figure 4B are boxed in yellow.
